# Asperosaponin VI ameliorates the CMS-induced depressive-like behaviors by inducing a neuroprotective microglial phenotype in hippocampus via PPAR-γ pathway

**DOI:** 10.1186/s12974-022-02478-y

**Published:** 2022-05-24

**Authors:** Xue Jiang, Saini Yi, Qin Liu, Dapeng Su, Liangyuan Li, Chenghong Xiao, Jinqiang Zhang

**Affiliations:** 1 Laboratory of Neuropharmacology, Resource Institute for Chinese and Ethnic Materia Medica, Guizhou University of Traditional Chinese Medicine, Guiyang, 550025 China; 2 State Key Laboratory of Quality Research in Chinese Medicine and Institute of Chinese Medical Sciences, University of Macau, Avenida da Universidade, Taipa, Macau, 999078 China

**Keywords:** Asperosaponin VI, Depression, Microglia, Hippocampus, PPAR-γ, Neuroinflammatory

## Abstract

**Background:**

The natural compound asperosaponin VI has shown potential as an antidepressant, but how it works is unclear. Here, we explored its effects on mice exposed to chronic mild stress (CMS) and the underlying molecular pathways.

**Methods:**

Mice were exposed to CMS for 3 weeks followed by asperosaponin VI (40 mg/kg) or imipramine (20 mg/kg) for another 3 weeks. Depression-like behaviors were assessed in the forced swimming test (FST), sucrose preference test (SPT), tail suspension test (TST). Microglial phenotypes were evaluated using immunofluorescence staining, real-time quantitative PCR and enzyme-linked immunosorbent assays in hippocampus of mice. In some experiments, stressed animals were treated with the PPAR-γ antagonist GW9662 to examine its involvement in the effects of asperosaponin VI. Blockade of PPAR-γ in asperosaponin VI-treated primary microglia in the presence of lipopolysaccharide (LPS) was executed synchronously. The nuclear transfer of PPAR-γ in microglia was detected by immunofluorescence staining in vitro and in vivo. A co-cultured model of neuron and microglia was used for evaluating the regulation of ASA VI on the microglia–neuron crosstalk molecules.

**Results:**

Asperosaponin VI ameliorated depression-like behaviors of CMS mice based on SPT, TST and FST, and this was associated with a switch of hippocampal microglia from a pro-inflammatory (iNOS^+^-Iba1^+^) to neuroprotective (Arg-1^+^-Iba1^+^) phenotype. CMS reduced the expression levels of PPAR-γ and phosphorylated PPAR-γ in hippocampus, which asperosaponin VI partially reversed. GW9662 treatment prevented the nuclear transfer of PPAR-γ in asperosaponin VI-treated microglia and inhibited the induction of Arg-1^+^ microglia. Blockade of PPAR-γ signaling also abolished the ability of asperosaponin VI to suppress pro-inflammatory cytokines while elevating anti-inflammatory cytokines in the hippocampus of CMS mice. The asperosaponin VI also promoted interactions between hippocampal microglia and neurons by enhancing CX3CL1/CX3CR1 and CD200/CD200R, and preserved synaptic function based on PSD95, CamKII β and GluA levels, but not in the presence of GW9662. Blockade of PPAR-γ signaling also abolished the antidepressant effects of asperosaponin VI in the SPT, TST and FST.

**Conclusion:**

CMS in mice induces a pro-inflammatory microglial phenotype that causes reduced crosstalk between microglia and neuron, inflammation and synaptic dysfunction in the hippocampus, ultimately leading to depression-like behaviors. Asperosaponin VI may ameliorate the effects of CMS by inducing microglia to adopt a PPAR-γ-dependent neuroprotective phenotype.

**Supplementary Information:**

The online version contains supplementary material available at 10.1186/s12974-022-02478-y.

## Introduction

Major depressive disorder (MDD) is a pervasive neuropsychiatric disorder and a significant contributor to the global burden of disease [1]. MDD has heterogeneous causes and clinical manifestations, which has impeded an understanding of its pathogenesis and design of effective treatments [2, 3]. Antidepressants based on monoamine neurotransmitters can reduce symptoms, but they are effective in only one-third to half of patients [4–6]. Even when effective, current drugs take 2–6 weeks to begin working, and they often produce adverse effects [7]. Therefore, developing more effective antidepressants with fewer side effects is urgently needed.

MDD is associated with neuroinflammation, such as the increased concentrations of pro-inflammatory cytokines [8, 9]. Such neuroinflammation can be driven by microglia, the innate immune cells of the central nervous system [10, 11]. Sustained microglial activation initiates a chronic neuroinflammatory response which can disturb neuronal health and disrupt communications between neurons and microglia [12]. Dysfunction of crosstalk between microglia and neuron caused by stress has a significant impact on the pathophysiology of depression [13]. When continuously stimulated by immune responses, microglia can adopt a pro-inflammatory phenotype, secreting pro-inflammatory cytokines and neurotoxic substances, which damage synapses, induce apoptosis, inhibit neurogenesis, and eventually lead to depression symptoms [14]. Conversely, certain signals can induce microglia to adopt a protective phenotype, in which scavenger receptors on their surface recognize metabolic wastes and nerve cell debris, which the microglia phagocytose [15, 16]. The microglia also releases anti-inflammatory cytokines and neurotrophic factors that protect and repair neurons, ultimately alleviating depression symptoms [17]. Therefore, regulating the phenotype of activated microglia is an attractive strategy for treating depression.

The triterpenoid saponin asperosaponin VI, one of the active components of the traditional Chinese medicine *Radix Dipsaci*, exerts anti-osteoporotic and anti-inflammatory effects, and it can pass through the blood–brain barrier to protect neurons and improve neurological diseases [18–22]. In previous work, we showed that asperosaponin VI, delivered at a dose of 40 mg/kg, can inhibit neuroinflammatory responses by hippocampal microglia and mitigate depression-like behaviors induced by lipopolysaccharide in mice [23]. These findings suggested that asperosaponin VI exerts its antidepressant effects at least in part by regulating hippocampal microglial function.

To verify and extend these previous findings, we examined the effects of asperosaponin VI on microglial phenotype and behaviors of mice exposed to chronic mild stress (CMS), a classical model of MDD. We also examined the potential involvement of the PPAR-γ pathway in mediating the effects of asperosaponin VI.

## Materials and methods

### Animals

Male 8-week-old C57BL/6 mice were purchased from Changsha Tianqin Biotechnology (Changsha, China) and allowed to acclimate for 1 week prior to experiments. All mice were housed individually under a standard 12-h light-to-dark cycle in temperature- and humidity-controlled rooms throughout the experiments. Mice were randomly assigned to experimental or control groups and an observer blinded to treatment conditions performed the behavioral tests and collected and analyzed the data. All experiments were approved by the Institutional Animal Care and Use Committee of the Guizhou University of Traditional Chinese Medicine.

### Chronic mild stress (CMS)

Mice 9 weeks old at the start of experiments were caged individually and subjected to CMS as described [17] for 3 weeks. Each day, animals were exposed to three of the following stressors: empty water bottles (12 h), food deprivation (12 h), tail clipping (10 min), restraint (2 h), lights-off for 3 h during the daylight phase, cage shaking (1 h), cage tilting (45°, 24 h), reversal of the light–dark cycle (24 h), strobe lighting (12 h), damp bedding (24 h) and a soiled cage (24 h). The mice were subjected to three different stressors per day at different times of the day to avoid habituation. The schedule is detailed in Additional file 1: Table S1.

### Treatment with asperosaponin VI or the PPAR-γ antagonist GW9662

Asperosaponin VI (99.92% pure; Chengdu Alfa Biotechnology Chengdu, China) was dissolved in 0.9% saline to a concentration of 2 mg/mL. After the 3-week CMS procedure, the mice were regrouped into five groups on the basis of the results of their sucrose preferences and body weights to ensure that there were no significant differences between the groups before the various treatments. After that, the mice received a single daily intraperitoneal injection of asperosaponin VI (40 mg/kg/d), imipramine (20 mg/kg/d; Sigma-Aldrich, St.Louis, MO, USA) or GW9662 (1 mg/kg; Sigma, St.Louis, MO, USA) for another 3 weeks. To examine the effects of asperosaponin VI on normal mice, 8-week-old male mice were received intraperitoneal injection with 0 to 200 mg/kg of asperosaponin VI for 7 consecutive days. One hour after the last injection, the spontaneous activity of the mice was measured by an open field test. The food consumption of the mice was checked daily.

### Behavioral testing

#### Sucrose preference test (SPT)

The SPT was performed as described [17]. Mice were individually housed, deprived of food and water for 12 h, and then given access to 1% sucrose solution (A) and water (B) for 2 h. The bottle positions were switched daily to avoid a side bias. The sucrose preference was calculated each week for each mouse using the formula: 100 × [VolA/(VolA + VolB)]. Sucrose consumption was normalized to the body weight of each mouse.

#### Tail suspension test (TST)

Each mouse was individually suspended by applying adhesive tape to the tip of the tail and connecting the tape to a ledge 30 cm above the cage floor. The animal was recorded for 6 min using a high-definition camera. An observer masked to treatment conditions recorded the latency between suspension and first abandonment of struggle as well as the time spent immobile during the 6-min period.

#### Forced swimming test (FST)

At 24 h before the test, mice were placed individually in a glass cylinder of height 25 cm and diameter 15 cm that was filled with water at 26 °C to a depth of 15 cm. The next day, the mice were placed again in the same situation and the immobility time was recorded for the last 4 min of the 6-min FST using a high-definition camera by an observer blinded.

#### Open field test (OFT)

Mice were placed into an open field (50 × 50 cm) and allowed to explore freely for 15 min. Total distance and time spent in the center (25 × 25 cm) were determined using video-tracking software (OFT100, Taimeng Tech. Chengdu, China).

#### Novelty-suppressed feeding test (NSFT)

Mice were deprived of food and water for 12 h before the test, then each mouse was placed for 5 min in a rectangular chamber (40 × 40 × 30 cm) containing a sugar pill in the center of the chamber. The time it took for a mouse to pick up the sugar with its forelimb was recorded as latency using a camera system.

### Cell culture and treatment

#### Primary culture of microglia and treatments

Primary microglia were isolated from brains of neonatal C57BL/6 mice (P0–P3) as described [15]. The purified microglial cells were cultured at 37 °C in DMED-F12 medium (Gibco, CA, USA) containing 10% fetal bovine serum (Gibco, CA, USA). Seven days later, microglia were pre-treated with 40 μM ASA VI (Alfabiotech, Chengdu, China) or 10 μM GW9662 (Sigma-Aldrich, MO, USA) [24]. After 30 min, microglia were treated for 24 h with either 50 ng/mL lipopolysaccharide (LPS; Sigma-Aldrich, MO, USA) or phosphate-buffered saline (PBS; BOSTER, Wuhan, China) (control). Following the immunocytochemistry, RT-PCR analysis and western blot analysis were performed.

#### Primary culture of hippocampal neurons

Primary neuron cultures were prepared as previously described [25]. In brief, the hippocampus were removed from postnatal day 0 (P0) male C57/BL/6J mice and dissociated cells were maintained in DMEM containing 10% fetal bovine serum (FBS, HyClone, Logan, UT, USA) and subsequently inoculated into a poly-d-lysine precoated 6-well plate at a density of 1 × 10^6^ cells/well. After 4 h of incubation, the culture medium was replaced with neurobasal medium (Gibco) with 2% B27 supplement (Gibco). Cultures were placed in a 37 °C humidified atmosphere with 5% CO_2_. Neurons were collected from days 5 to 7 of in vitro culture were used for the experiments.

#### Co-culture of microglia with neurons

Co-culture of microglia with neurons was performed using Transwell plate (Corning Company, NY, USA) as previously described [26]. Primary microglia were plated in the lower chamber of the Transwell plate at a density of 1 × 10^5^ cells/well. The microglia were pre-treated with ASA VI (40 μM) or GW9662 (10 μM) for 30 min, following LPS (50 ng/mL) or PBS administration. And then, neurons were plated in the upper chamber of Transwell plates at a density of 1 × 10^5^ cells/cm^2^. Plates were incubated at 37 °C in an atmosphere of 5% CO_2_ for 24 h.

### RNA extraction and real-time PCR

At the end of experiments, mice were perfused with 0.9% NaCl, the whole brain was removed, and the hippocampus and cortex were isolated and placed into separate enzyme-free 1.5-mL microcentrifuge tubes. Total RNA was extracted separately from primary microglia, the hippocampus and cortex of mice using Trizol (Invitrogen Life Technologies, Shanghai, China), then reverse-transcribed into cDNA using the high-capacity cDNA conversion kit (Takara, Tokyo, Japan) in strict accordance with the manufacturer’s instructions. RT-PCR reaction mixture contains 1 μg of template cDNA, 5 μL MasterMix, and 1 μL primer (Sangon Biotech, Sichuan, China); add DEPC water to a total reaction volume of 10 μL. The PCR was performed in a CFX 96 system (Bio-Rad, Hercules, California, USA) using the following steps: pre-denaturation at 95 °C for 30 s, denaturation at 95 °C for 5 s, then 39 cycles of annealing at the appropriate temperature for 34 s, followed by extension. Each sample was analyzed in three replicates. Expression level was determined using the 2^−ΔΔCt^ method with reference to the β-actin gene. The primers of each gene are listed in Additional file 1: Table S2.

### Immunocytochemistry

Whole brain from mice was perfused and fixed in 4% paraformaldehyde for 48 h, dehydrated, frozen, cut into thin sections, thoroughly cleaned with 0.5% Triton X-100 for 15 min, blocked with 10% donkey serum for 1 h, and incubated overnight at 4 °C with the following primary antibodies as listed in Additional file 1: Table S3. On the next day, slices were washed three times with phosphate-buffered saline (PBS) and incubated in the dark for 2 h with secondary antibodies (1:500; Jackson ImmunoResearch, West Grove, PA, USA). DAPI (1:10,000; Roche, Switzerland) was added to stain the nuclei, and slices were observed under a fluorescence microscope (Olympus BX 51, Japan). Images were imported into Image J software (version 1.45J; National Institutes of Health, Bethesda, MD), and an intensity threshold was defined to differentiate positive staining from background.

### Enzyme-linked immunosorbent assay (ELISA)

Hippocampus and cortex from mice were placed into separate 1.5-mL microcentrifuge tubes and completely homogenized. The concentration of total protein in the supernatant was determined using the BCA kit (Boster, Shanghai, China), then aliquots of the supernatants were diluted to the same total protein concentration. These diluted samples were assayed for interleukin (IL)-1β, IL-10 and brain-derived neurotrophic factor (BDNF) using a commercial ELISA kit (Boster, Shanghai, China) in strict accordance with the manufacturer’s instructions.

### Western blotting

The hippocampus and cortex of the mice were lysed by RIPA lysis buffer (Solarbio, Beijing, China) then centrifuged at 1000×*g* for 30 min. The concentration of total protein was measured by the BCA method. Equal amount of protein was resolved using 12% SDS polyacrylamide gel. Fractionated proteins were transferred onto PVDF membranes at 300 mA for 30 min, then the membrane was washed in TBST, blocked in skim milk for 30 min, and incubated overnight on a shaker at 4 °C with primary antibody as listed in Additional file 1: Table S4. The membrane was again washed three times with TBST, incubated with secondary antibody (1:10,000; Abcam, FCE, UK) for 30 min, washed three times with TBST, and Bands were visualized using the BM Chemiluminescence Western Blotting Kit (Roche Diagnostics GmbH, Mannheim, Germany). Membranes were analyzed using the ChemiDoc Touch system (Bio-Rad, Hercules, California, USA), and band intensity was quantified using Alpha software (version 1.45J; National Institutes of Health, Bethesda, MD, USA).

### Statistical analysis

All statistical analyses were performed using GraphPad Prism software (version 8.0, SPSS Inc., Chicago, USA). Data were presented as mean ± SEM. Pairwise comparisons were assessed for significance using Student’s two-tailed *t*-test, and comparisons among three or more values were assessed using one- or two-way ANOVA and Tukey’s post hoc tests. Differences were considered statically significant if *p* < 0.05.

## Results

### Asperosaponin VI ameliorates depression-like behaviors induced by CMS in mice

**Fig. 1:**
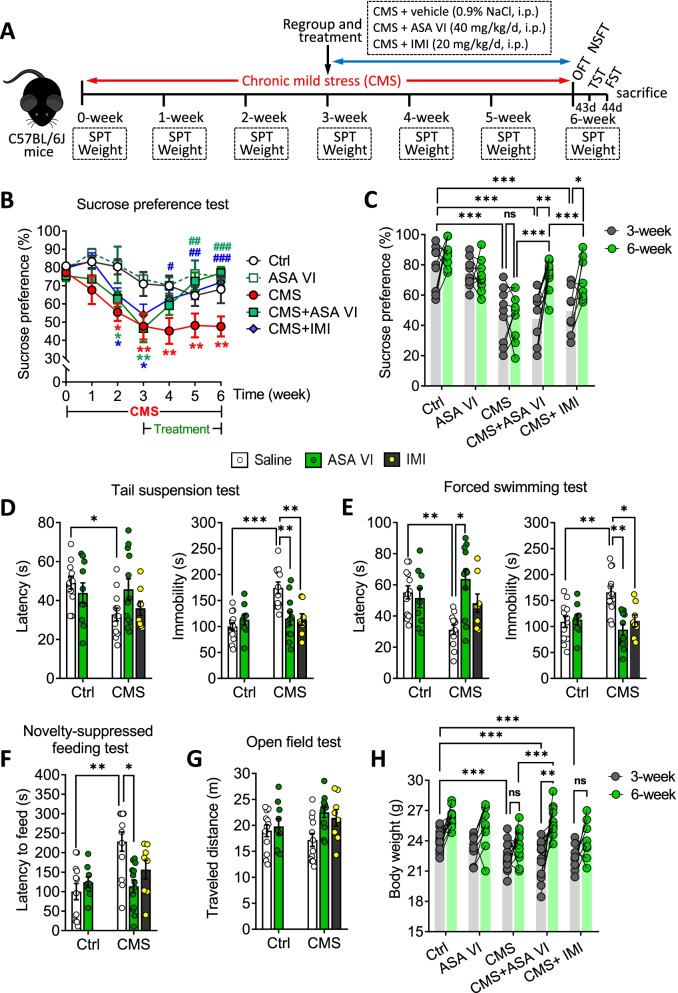
Asperosaponin VI ameliorates CMS-induced depression-like behaviors. **A** Timeline of the experimental process. **B** Sucrose preference during 6 weeks in Ctrl mice and mice subjected to CMS, followed by treatment with ASA VI or IMI. *p < 0.05, **p < 0.01 vs Ctrl group, ^#^*p* < 0.05, ^##^*p* < 0.01, ^###^*p* < 0.001 vs CMS group. **C** Sucrose preference of individual mice before treatment (3-week) or after treatment (6-week) with ASA VI or IMI. **D** Latency and time spent immobile in the TST. **E** Latency and time spent immobile in the FST. **F** Latency to feed in the NSFT. **G** Distance travelled in the OFT. **H** Body weight of Ctrl or CMS mice before treatment (3-week) or after treatment (6-week) with ASA VI or IMI. Data for individual animals are displayed (mean ± SEM, *n* = 8–12 per group). *p < 0.05, **p < 0.01, ***p < 0.001 (two-way ANOVA with Tukey's multiple-comparisons test). ASA VI, asperosaponin VI; CMS, chronic mild stress; Ctrl, the control; IMI, imipramine; TST, tail suspension test; FST, forced swimming test; NSFT, novelty-suppressed feeding test; OFT, open field test

Mice were exposed to CMS for 3 weeks and received a subsequent 3 weeks of imipramine or asperosaponin VI treatment (Fig. 1A). The results showed that sustained CMS suppressed the sucrose preference of the mice. However, imipramine and asperosaponin VI reversed this trend and returned the sucrose preference to normal levels by the third week of treatment (Fig. 1B). Analysis at the level of individual mice showed that there were no significant differences in the sucrose preferences between the groups before the various treatments. Treatment with 40 mg/kg asperosaponin VI for 3 weeks improved sucrose preference in nearly 90% of CMS mice, while imipramine improved it in just over 60% (Fig. 1C).

CMS shortened latency and led to longer time spent immobile in the TST (Fig. 1D) and FST (Fig. 1E). Asperosaponin VI, but not imipramine, prolonged latency in the FST and feeding latency in the NSFT, while both compounds shortened the time spent immobile in the TST and FST (Fig. 1D–F). Neither compound affected the distance travelled in the OFT (Fig. 1G), indicating that asperosaponin VI does not affect nerve transmission. Asperosaponin VI partially reversed the weight loss induced by CMS (Fig. 1H).

### Asperosaponin VI induces hippocampal microglia to switch from a pro-inflammatory to anti-inflammatory phenotype after CMS

**Fig. 2:**
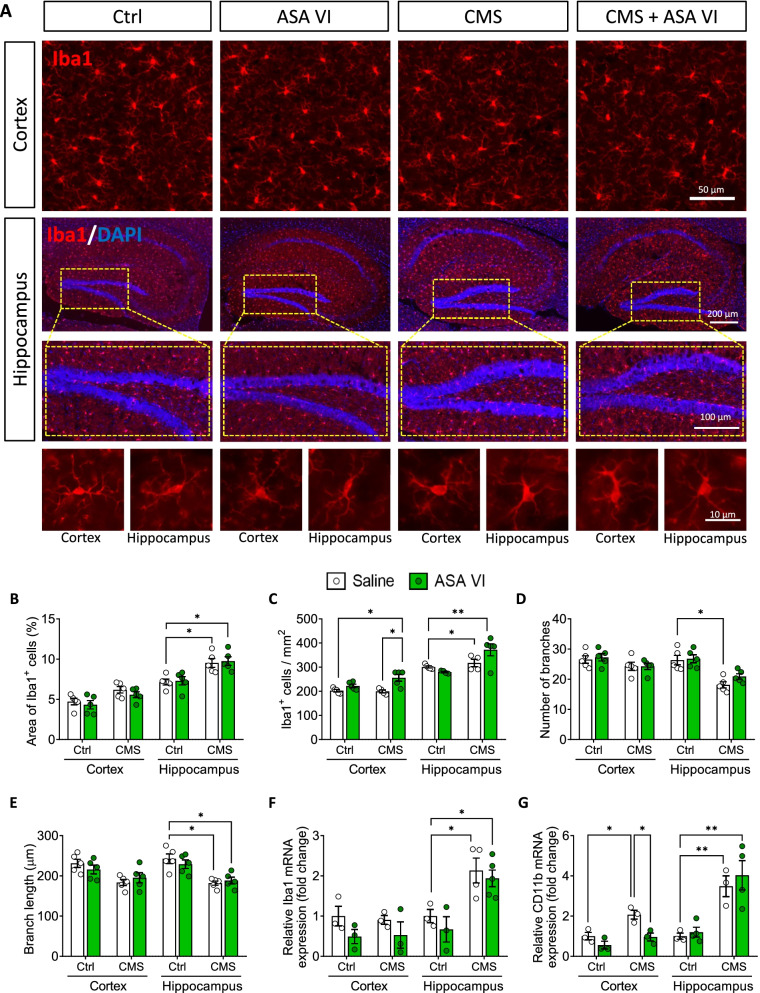
Effects of asperosaponin VI on the morphology of microglia in hippocampus of CMS mice. **A** Representative micrographs of microglia in hippocampus and cortex of Ctrl or CMS mice after treatment with saline or ASA VI. Microglia were stained with Iba1 (red) by immunocytochemistry, and nuclei were stained with DAPI (blue). **B**–**E** Quantification of the area and number of Iba1^+^ cells, number and length of microglial branches in hippocampus and cortex of Ctrl or CMS mice after treatment with saline or ASA VI. Results of each group were obtained from 5 mice, and 5–6 micrographs (40 ×) containing hippocampus were collected from each sample. All Iba1^+^ cells in each micrograph were measured. Each dot in the bar graph represents the average of all Iba1^+^ cells analyzed for each mouse. **F** and **G** Levels of mRNA encoding Iba1 and CD11b in hippocampus and cortex of Ctrl or CMS mice after treatment with saline or ASA VI (n = 4 animals). Data for individual animals are displayed (mean ± SEM), *p < 0.05, **p < 0.01 (two-way ANOVA with Tukey’s multiple-comparisons test)

Microscopy of tissue slices showed that CMS induced soma enlargement, thickening and shortening of processes and loss of branching in hippocampal microglia (Fig. 2A–E). CMS also increased expression of the microglial marker Iba1 and the marker of microglial activation CD11b in the hippocampus, as well as expression of CD11b in cortex (Fig. 2F and G). These findings indicate stress-induced microglial activation.

**Fig. 3:**
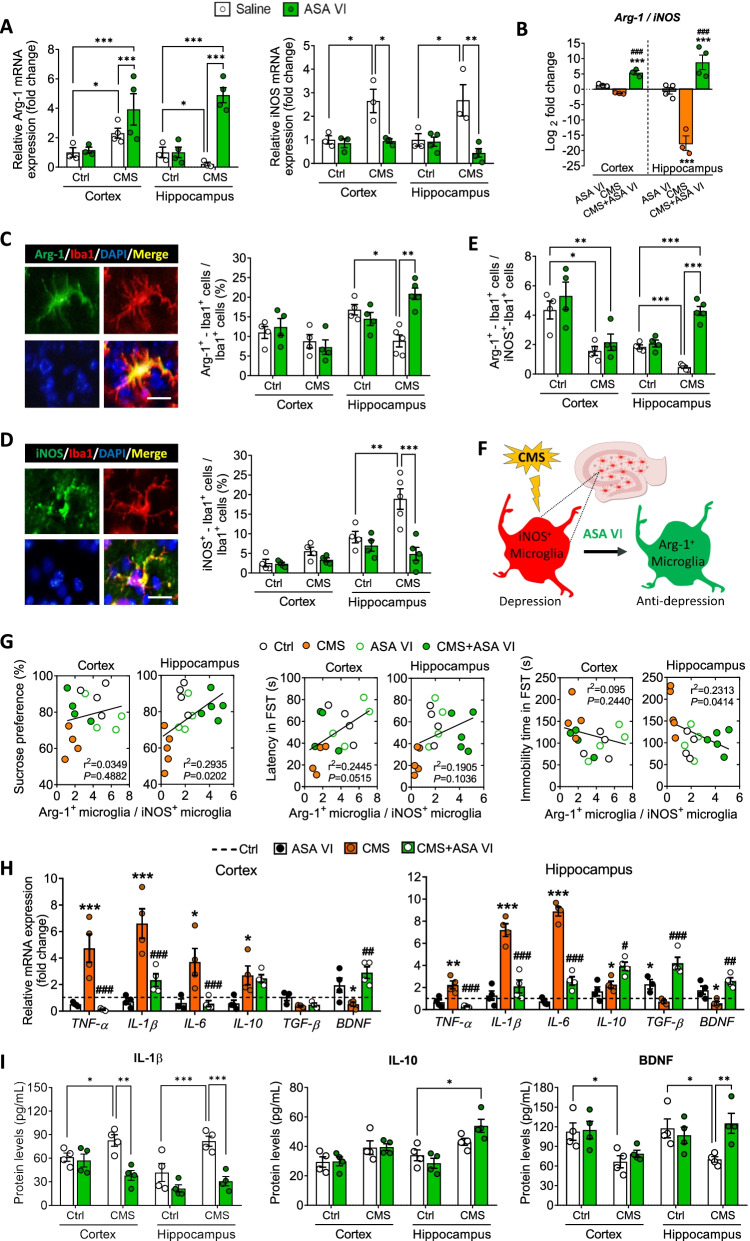
Asperosaponin VI switches activated microglia from a pro-inflammatory to anti-inflammatory phenotype in hippocampus of CMS mice. **A** Levels of mRNAs encoding Arg-1 and iNOS in hippocampus and cortex of Ctrl or CMS mice after treatment with saline or ASA VI. **B** Ratio of the levels of mRNAs encoding Arg-1 to iNOS in hippocampus and cortex of Ctrl or CMS mice after treatment with saline or ASA VI. **C** Fluorescence micrographs of anti-inflammatory microglia (Arg-1^+^-Iba1^+^ cells) in hippocampus of CMS mice after treatment with ASA VI. Anti-inflammatory marker was stained with an antibody against Arg-1 (green); microglia, with an antibody against Iba1 (red); and nuclei, with DAPI (blue). The cells positive for Arg-1 and Iba1 were considered anti-inflammatory microglia. Histogram is quantification of the percentage of Arg-1^+^-Iba1^+^ cells. Scale bar, 10 μm. **D** Fluorescence micrographs of pro-inflammatory microglia (iNOS^+^-Iba1^+^ cells) in hippocampus of CMS mice. Pro-inflammatory cytokines were stained with an antibody against iNOS (green). The cells positive for iNOS and Iba1 were considered pro-inflammatory microglia. Histogram is the quantification of the percentage of iNOS^+^-Iba1^+^ cells. Scale bar, 10 μm. **E** Ratio of Arg-1^+^ microglia to iNOS^+^ microglia in hippocampus and cortex of Ctrl or CMS mice after treatment with saline or ASA VI. **F** Schematic illustrating how ASA VI switches activated microglia from a pro-inflammatory to anti-inflammatory phenotype in hippocampus of CMS mice. **G** Correlation of the ratio of Arg-1^+^ microglia to iNOS^+^ microglia in hippocampus or cortex of each group mice with sucrose preference and latency and immobility time in the FST. **H** Levels of mRNAs encoding the pro-inflammatory cytokines (TNF-α, IL-1β, and IL-6) and the anti-inflammatory cytokines (IL-10 and TGF-β) and brain-derived neurotrophic factor (BDNF) in hippocampus and cortex of Ctrl or CMS mice after treatment with saline or ASA VI. **I** Levels of IL-1β, IL-10 and BDNF in hippocampus and cortex of Ctrl or CMS mice after treatment with saline or ASA VI. Data for individual animals are displayed (mean ± SEM, *n* = 3–5), **A**, **C**–**E** and **I** *p < 0.05, **p < 0.01, ***p < 0.001. **B** and **H** *p < 0.05, *p < 0.01, ***p < 0.001 vs. Ctrl group, ^#^*p* < 0.05, ^##^*p* < 0.01, ^###^*p* < 0.001 vs. CMS group (two-way ANOVA with Tukey's multiple-comparisons test)

Asperosaponin VI did not cause obvious changes in morphology or CD11b expression in microglia in the hippocampus or cortex, nor did it reverse the increase in Iba1^+^ area, increase in cell number or decrease in microglial branching induced by CMS (Fig. 2A–G). Similarly, the compound did not alter the CMS-induced radial morphology of astrocytes in the hippocampus and cortex (Additional file 1: Fig. S1 and S2). These results suggest that asperosaponin VI does not inhibit CMS-induced activation of hippocampal microglia; instead, it may regulate the type of microglial activation to influence depression-like behaviors. Consistent with this hypothesis, we found that CMS decreased Arg-1 expression and the proportions of anti-inflammatory (Arg-1^+^-Iba1^+^) in hippocampus but not in cortex and increased iNOS expression and the proportions of pro-inflammatory (iNOS^+^-Iba1^+^) microglia (Fig. 3A–D). Asperosaponin VI partially reversed these changes and significantly elevated the ratio of Arg-1^+^ microglia to iNOS^+^ microglia in hippocampus but not cortex of CMS mice (Fig. 3E). These results suggest that asperosaponin VI switches the phenotype of activated microglia in hippocampus from pro-inflammatory (iNOS^+^) to neuroprotective (Arg-1^+^), which may be involved in the antidepressant process of asperosaponin VI (Fig. 3F). Consistent with this hypothesis, we found that the ratio of Arg-1^+^ microglia to iNOS^+^ microglia in hippocampus but not cortex was positively correlated with sucrose preference and negatively correlated with immobility time in FST (Fig. 3G).

Similarly, CMS increased levels of the pro-inflammatory cytokines IL-1β, IL-6 and TNF-α in cortex and hippocampus (Fig. 3H and I). Asperosaponin VI partially reversed these changes while also upregulating the protective molecules interleukin (IL)-10, transforming growth factor (TGF)-β and BDNF in hippocampus (Fig. 3H and I).

### Asperosaponin VI acts via the PPAR-γ pathway to exert its anti-inflammatory and antidepressant effects

**Fig. 4:**
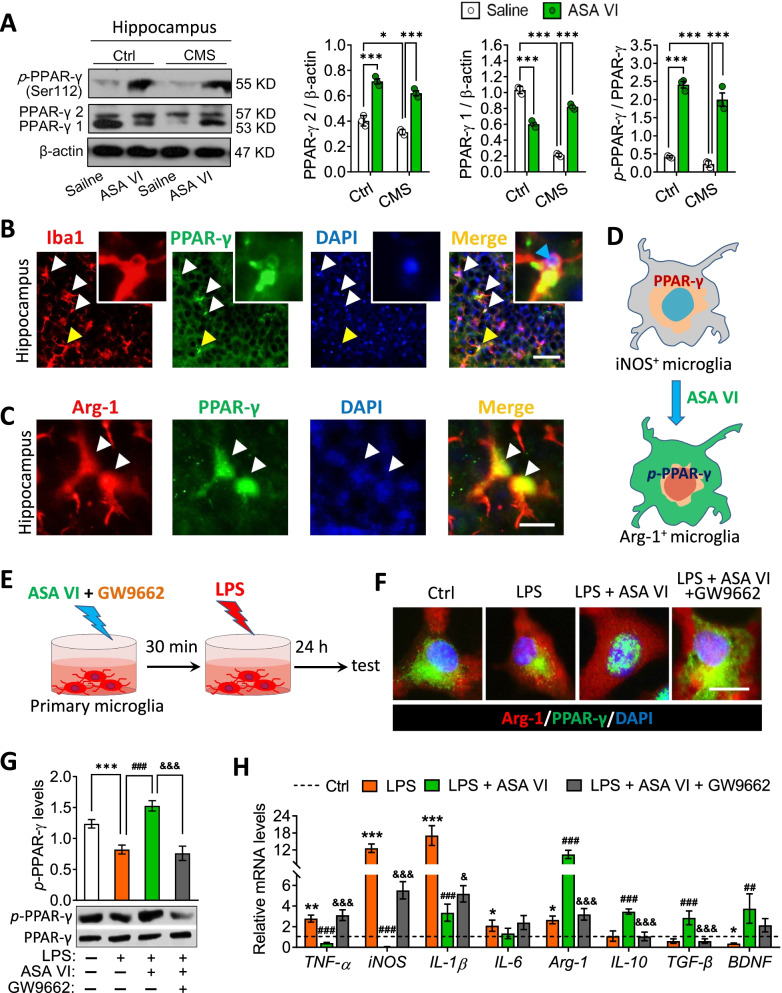
Asperosaponin VI activates the PPAR-γ in hippocampal microglia of CMS mice and in primary microglia. **A** Levels of *p*-PPAR-γ, PPAR-γ 1 and PPAR-γ 2 in hippocampus of Ctrl or CMS mice after treatment with saline or ASA VI. Levels of PPAR-γ-1 and PPAR-γ-2 were normalized to those of β-actin. Levels of phosphorylated PPAR-γ were normalized to those of PPAR-γ-1 and PPAR-γ-2 (*n* = 3, each sample in triplicate). **B** Fluorescence micrographs showing PPAR-γ expression in microglia (as indicated by the white arrow) of hippocampus in CMS mice after treatment with ASA VI. PPAR-γ was stained with antibody (green), microglia were stained with an anti-Iba1 antibody (red), and nuclei were stained with DAPI (blue). The yellow arrow indicates the high-resolution cells inserted in the upper right. The blue arrow indicates that the nuclear transfer of PPAR-γ is in the hippocampal microglia. Scale bar, 20 μm. **C** Fluorescence micrographs showing PPAR-γ expression in Arg-1^+^ cells (as indicated by the white arrow) of hippocampus in CMS mice after treatment with ASA VI. Scale bar, 10 μm. **D** Schematic illustrating ASA VI switches activated microglia from a pro-inflammatory (iNOS^+^) to anti-inflammatory (Arg-1^+^) phenotype via the PPAR-γ activation in hippocampus of CMS mice. **E** Experimental scheme to monitor the effect of GW9662 on PPAR-γ in Ctrl, lipopolysaccharide (LPS), LPS + ASA VI and LPS + ASA VI + GW9662 in primary microglia. **F** Fluorescence micrographs showing nuclear translocation of PPAR-γ in Ctrl, LPS, LPS + ASA VI and LPS + ASA VI + GW9662 microglia. PPAR-γ was stained with PPAR-γ antibody (green), Arg-1 in microglia were stained with an anti-Arg-1 antibody (red), and nuclei were stained with DAPI (blue). Scale bar, 10 μm. **G** Effects of GW9662 treatment on levels of *p*-PPAR-γ in Ctrl, LPS, LPS + ASA VI and LPS + ASA VI + GW9662 microglia (*n* = 3, each sample in triplicate). **H** Levels of mRNAs encoding the pro-inflammatory cytokines (TNF-α, iNOS, IL-1β, and IL-6) and the anti-inflammatory cytokines (Arg-1, IL-10 and TGF-β) and BDNF in Ctrl, LPS, LPS + ASA VI and LPS + ASA VI + GW9662 microglia. Data are displayed with mean ± SEM (*n* = 3–5), **A** *p < 0.05, ***p < 0.001 (two-way ANOVA with Tukey’s multiple-comparisons test). **G** and **H** *p < 0.05, *p < 0.01, ***p < 0.001 vs. Ctrl group, ^#^*p* < 0.05, ^##^*p* < 0.01, ^###^*p* < 0.001 vs. LPS group, ^&^*p* < 0.05, ^&&&^*p* < 0.001 vs. LPS + ASA VI group (one-way ANOVA with Tukey’s multiple-comparisons test)

Since PPAR-γ signaling plays a key role in anti-inflammatory microglial phenotypes, we asked whether asperosaponin VI acts via such signaling to exert its “phenotype switching” effect. Indeed, CMS reduced expression of PPAR-γ-1 and PPAR-γ-2 as well as levels of phosphorylated PPAR-γ in hippocampus, which asperosaponin VI partially reversed (Fig. 4A). PPAR-γ localized in cytoplasm and nucleus of microglia and Arg-1^+^ cells in the hippocampus of mice that were exposed to CMS and then treated with asperosaponin VI (Fig. 4B and C).

We conclude that asperosaponin VI-induced Arg-1^+^ microglia be driven by nuclear transfer of PPAR-γ under adverse condition (4D). Based on this, we blocked the PPAR-γ pathway in asperosaponin VI-treated microglia in the presence of lipopolysaccharide (LPS) using the PPAR-γ antagonist GW9662 (4E). The result showed that GW9662 treatment prevented the nuclear transfer of PPAR-γ and suppressed the Arg-1 expression in asperosaponin VI-treated microglia (Fig. 4F and G). Blockade of PPAR-γ signaling also abolished the ability of asperosaponin VI to suppress pro-inflammatory cytokines and elevate anti-inflammatory cytokines in the LPS-exposed microglia (Fig. 4H).

**Fig. 5:**
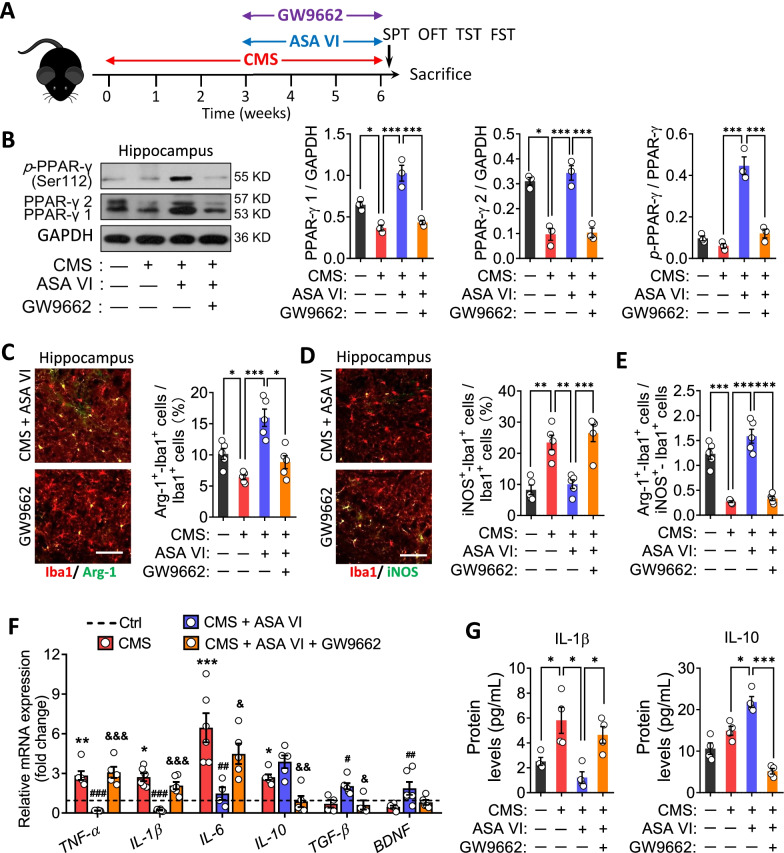
Asperosaponin VI induces an anti-inflammatory microglial phenotype via PPAR-γ. **A** Timeline of the experimental process on the effect of GW9662 on ASA VI-treated CMS mice. **B** Effects of GW9662 treatment on levels of p-PPAR-γ, PPAR-γ-1, and PPAR-γ-2 in hippocampus of ASA VI + CMS mice (*n* = 3, each sample in triplicate). **C** and **D** Effects of GW9662 treatment on microglia with pro- or anti-inflammatory phenotypes in hippocampus of ASA VI + CMS mice. Scale bar, 50 μm. **E** Effects of GW9662 treatment on the ratio of Arg-1^+^ microglia to iNOS^+^ microglia in hippocampus of ASA VI + CMS mice. **F** Effects of GW9662 treatment on mRNA levels of pro-inflammatory cytokines (TNF-α, IL-1β, and IL-6), anti-inflammatory cytokines (IL-10 and TGF-β) and BDNF. **G** Effects of GW9662 treatment on protein levels of IL-1β and IL-10 in hippocampus of ASA VI + CMS mice. Data for individual animals are displayed (mean ± SEM, *n* = 3–6), **B**–**E** and **G** *p < 0.05, **p < 0.01, ***p < 0.001 (one-way ANOVA with Tukey’s multiple-comparisons test). **F** *p < 0.05, **p < 0.01, ***p < 0.001 vs. Ctrl group, ^#^*p* < 0.05, ^##^*p* < 0.01 vs. CMS group, ^&^*p* < 0.05, ^&&^*p* < 0.01, ^&&&^*p* < 0.001 vs. CMS + ASA VI group (one-way ANOVA with Tukey’s multiple-comparisons test)

To confirm the role of PPAR-γ in mediating the anti-inflammatory effects of asperosaponin VI, we repeated the above experiments in the presence of the PPAR-γ antagonist GW9662 (Fig. 5A), which effectively blocked the PPAR-γ pathway in hippocampus (Fig. 5B). Such blockade abolished ability of asperosaponin VI to increase numbers of Arg-1^+^ microglia and decrease numbers of iNOS^+^ microglia in the hippocampus of CMS mice (Fig. 5C–E). Blockade of PPAR-γ signaling also abolished the ability of asperosaponin VI to suppress pro-inflammatory cytokines and elevate anti-inflammatory cytokines in the hippocampus of CMS mice (Fig. 5F and G). These results suggest that asperosaponin VI exerts its anti-inflammatory effects via the PPAR-γ signaling pathway.

### Asperosaponin VI promotes microglia–neuron crosstalk and prevents the CMS-induced synaptic dysfunction of hippocampal neurons via a PPAR-γ-dependent pathway

**Fig. 6:**
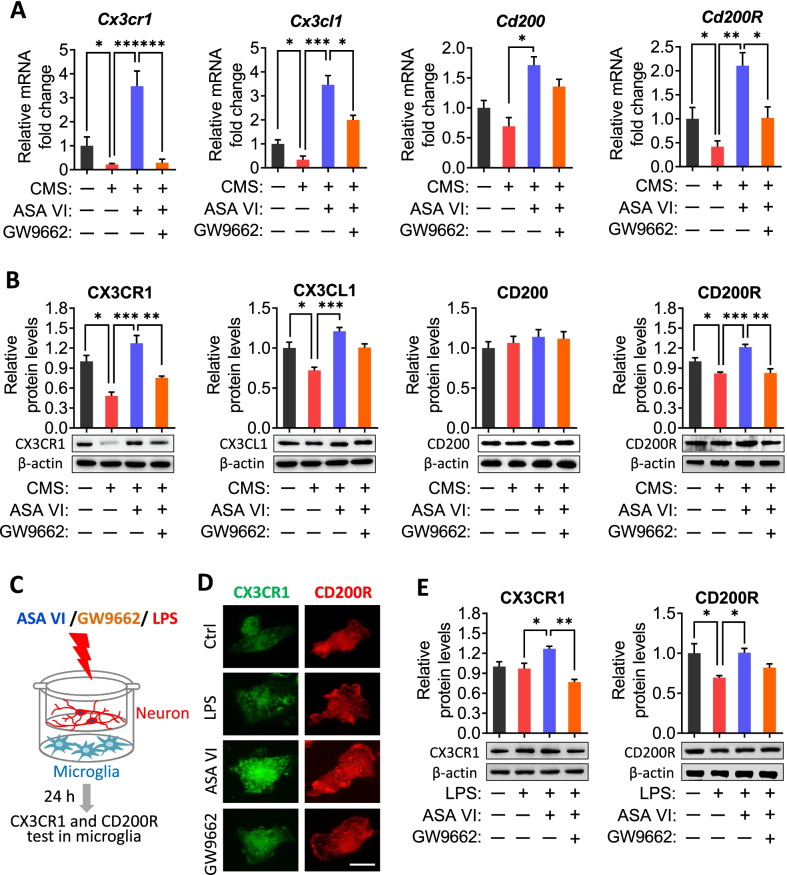
Asperosaponin VI restores the microglia–neuron crosstalk in vivo and in vitro. **A** and **B** Effects of GW9662 treatment on mRNA and protein levels of ligand-receptor pairs that mediate crosstalk between neurons and microglia (Cx3CR1/Cx3CL1, CD200/CD200R) in hippocampus of ASA VI + CMS mice. **C** Schematic diagram of the neuron–microglia cell co-culture system. Neurons were seeded in the top compartment of the transwell, and microglia were cultured in the bottom compartment. Subsequently, the co-cultured cells were treated with PBS or LPS for 24 h in presence or absence ASA VI or/and GW9662. **D** and **E** The effects of GW9662 treatment on endogenous CX3CR1 and CD200R expression in primary microglia by immunofluorescence staining and western blotting after the microglia co-cultured with neuron were treat with LPS for 24 h in presence ASA VI. Scale bar, 5 μm. Data are displayed with mean ± SEM (*n* = 4–5), *p < 0.05, **p < 0.01, ***p < 0.001 (one-way ANOVA with Tukey’s multiple-comparisons test)

Dysfunctional microglia can communicate abnormally with neurons, which disrupts synaptic function and may help to explain MDD behaviors. In our mice, CMS reduced hippocampal expression of CX3CL1 and its receptor CX3CR1 as well as expression of CD200 and its receptor CD200R; these pairs mediate communication between neurons and microglia. Asperosaponin VI partially reversed these CMS-induced changes, while GW9662 abolished the effects of asperosaponin VI (Fig. 6A and B). To further clarify whether asperosaponin VI directly regulates the microglia–neuron crosstalk, we established a co-cultured model of neuron and microglia (Fig. 6C). The results showed that asperosaponin VI upregulated the CX3CR1 expression and restored CD200R expression in LPS-treated microglia that these effects were prevented by GW9662 treatment (Fig. 6D and E).

**Fig. 7:**
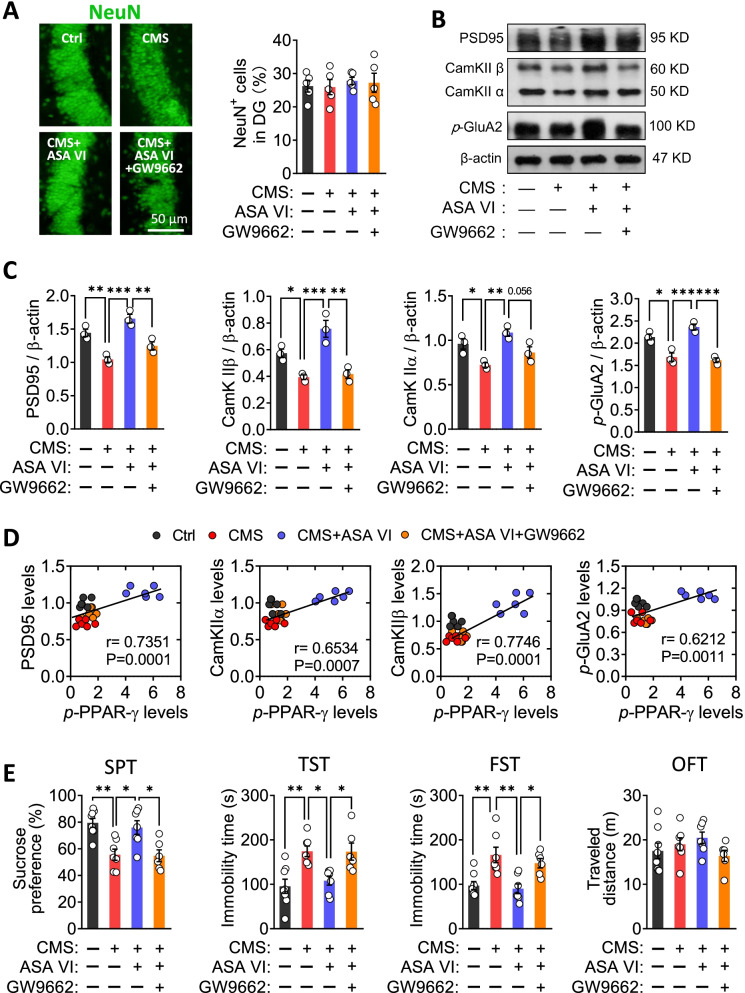
Asperosaponin VI prevents the CMS-induced synaptic dysfunction of hippocampal neurons via a PPAR-γ-dependent pathway. **A** Fluorescence micrographs of mature neurons in the DG of hippocampus, identified through staining for NeuN. At right is quantification of the area of NeuN^+^ cells in the DG. Each dot in the bar graph represents the average of 5–6 micrographs for each mouse (*n* = 5). **B** and **C** Effects of asperosaponin VI on levels of PSD95, CamKII α, CamKII β, and p-GluA2 in hippocampus of CMS mice treated with GWP662 or not. Levels of proteins were normalized to those of β-actin (*n* = 3, each sample in triplicate). **D** Correlation of the levels of *p*-PPA-γ with the levels of PSD-95, CamKII α, CamKII β, and p-GluA2. **E** Effects of GW9662 treatment on the sucrose preference (SPT), immobility time in tail suspension test (TST) and forced swimming test (FST) and the traveled distance in open field test (OFT) of ASA VI + CMS mice (*n* = 7–9). Data for individual animals are displayed (mean ± SEM), *p < 0.05, **p < 0.01, ***p < 0.005 (one-way ANOVA with Tukey’s multiple-comparisons test)

Abnormal interactions between microglia and neuron in hippocampus will lead to synaptic dysfunction which link to depressive symptoms. Indeed, our results showed that CMS downregulated PSD95, CamKII α and CamKII β as well as decreased levels of phosphorylated GluA 2 in hippocampus, but did not affect the area of NeuN^+^ cells in the hippocampal dentate gyrus, all of which suggest inhibition of synaptic function but not apoptosis of mature neurons (Fig. 7A–C). These changes in synaptic function-related markers were partially reversed by asperosaponin VI, but not in the presence of GW9662 (Fig. 7B and C). Levels of PPAR-γ positively correlated with levels of PSD95, CamKII α, CamKII β and phosphorylated GluA 2 (Fig. 7D). These results suggest that asperosaponin VI requires PPAR-γ to induce neuroprotective microglia and repair CMS-induced damage to synaptic function in hippocampus. Blockade of PPAR-γ signaling also abolished the antidepressant effects of asperosaponin VI in the SPT, TST and FST (Fig. 7E). These results suggest that asperosaponin VI exerts its antidepressant via the PPAR-γ signaling pathway.

## Discussion

Regulating the phenotype of activated microglia is an attractive strategy for treating depression. Here, using a classical animal model of depression, we showed that asperosaponin VI induces a PPAR-γ-dependent neuroprotective microglial phenotype that mitigates depression-like behaviors induced by CMS while possibly contributing to microglia–neuron crosstalk. Our work extends the list of conditions where asperosaponin VI can exert therapeutic anti-inflammatory and neuroprotective effects in the brain, a list that already includes Alzheimer’s disease and optic nerve damage.

Depression usually manifests as diverse debilitating symptoms, including hopelessness and anhedonia [27]. Anhedonia, a core symptom of MDD, can be assessed in the SPT [28]. In addition to the SPT, we used the FST and TST to assess passive stress-coping behavioral despair [29, 30]. As expected, CMS caused depression-like behaviors in all these tests, which subsequent asperosaponin VI treatment improved, to an even greater extent than the classic monoamine antidepressant imipramine. In fact, asperosaponin VI but not imipramine partially restored weight loss caused by CMS, suggesting that the former may lack serious side effects at the dose of 40 mg/kg. We believe that these results indicate antidepressant-like effects of asperosaponin VI, because the compound did not significantly alter performance in the OFT.

The dysregulation of pro- and anti-inflammatory cytokines plays a crucial role in depression [31–33]. In the present study, CMS upregulated the pro-inflammatory cytokines IL-1β, IL-6, iNOS and TNF-α in hippocampus of mice, and asperosaponin VI reversed these changes while also upregulating the anti-inflammatory cytokines Arg-1, IL-10, and TGF-β as well as BDNF. These results establish a link between the neuroprotective and anti-inflammatory effects of asperosaponin VI. The hippocampus is part of the limbic system and develops nerve fiber connectivity with emotion-related brain regions, for instance, the prefrontal cortex and amygdala [34]. Studies showed that microglia are activated more easily in the hippocampus than in other brain regions [10, 35]. And this may explain in part what we found in this study that inflammatory factors in the cortex are not as significant as they are in the hippocampus. Astrocytes have also been shown to play an important role in depression and neuroinflammation [36]. In this study, we found the number or area of neither GFAP^+^ cells nor S100 β^+^ cells were not changed in each group. However, several studies have found that astrocyte function is altered in CMS mice and contributes to the pathology of depression [37]. This also indirectly reflects the astrocytes do not correspond in shape and function as microglia do. The role of asperosaponin VI in regulating astrocyte function and its role in antidepressant should be further studied.

Stress or immunostimulation has been shown to induce neuroinflammation, which appears to involve microglial activation, particularly in the hippocampus [38, 39]. Consistent with these previous studies, we found here that CMS caused morphological changes in hippocampal microglia indicative of microglial activation. Asperosaponin VI did not reduce the *extent* of overall microglial activation in hippocampus of CMS mice; instead, it altered the *type* of such activation, from a pro-inflammatory to neuroprotective phenotype. The decrease in proportion of pro-inflammatory microglia translates to lower production of pro-inflammatory cytokines and neurotoxic products (such as nitric oxide and quinolinic acid) [40], which increase neuropathic pain and inhibit hippocampal neurogenesis, contributing to cognitive deficits and depression-like behaviors [10, 41–43]. Arg1^+^ microglia can be thought of a special phenotype of beneficial microglia. These cells regulate inflammatory processes in the brain and enhance neurite growth, thereby exhibiting neuroprotective effects [44–46]. Studies demonstrated that Arg1^+^ microglia reduce inflammation and promote neurogenesis in the hippocampus of CMS-exposed mice by reducing the synthesis and secretion of pro-inflammatory mediators and increasing BDNF expression [15, 47]. In this study, we also found asperosaponin VI treatment increased the number of Arg1^+^ microglia and BDNF expression in hippocampus of CMS mice. These findings support the idea that the asperosaponin VI induces a neuroprotective phenotype of microglia in hippocampus of CMS-exposed mice.

We found that the anti-inflammatory effects of asperosaponin VI are mediated by PPAR-γ, a ligand-dependent transcription factor belonging to the nuclear hormone receptor superfamily [48]. PPAR-γ regulates the expression of anti-inflammatory cytokines [49], and the PPAR-γ agonists pioglitazone or rosiglitazone can switch activated microglia cells from a pro-inflammatory to anti-inflammatory state [50, 51]. Previous research showed that asperosaponin VI acts via PPAR-γ to switch activated microglia from a pro-inflammatory to anti-inflammatory phenotype in vitro [52]. In present study, we further demonstrated that asperosaponin VI acts via PPAR-γ to induce a neuroprotective phenotype in hippocampal microglia of CMS-exposed mice and mitigate depressive-like mouse behaviors. Conversely, blocking the PPAR-γ signaling pathway abolished the neuroprotective microglia induced by asperosaponin VI in hippocampus of CMS-exposed mice, as well as the antidepressant effect of asperosaponin VI. Thus, we speculated that asperosaponin VI exerts its antidepressant and anti-inflammatory effects via the PPAR-γ signaling pathway to regulate the phenotype of microglia.

The continuous crosstalk between microglia and neurons is required for microglia housekeeping functions and contributes to brain homeostasis. These interactions lay on the delicate edge between physiological processes and homeostasis alteration in pathology and are themselves altered during neuroinflammation [13, 53, 54]. Sustained microglial activation initiates a chronic neuroinflammatory response which can disturb neuronal health and disrupt communications between neurons and microglia [12]. The CX3CL1/CX3CR1 and CD200/CD200R axis were the immune checkpoint of neuron–microglia crosstalk, through which neurons suppress microglial overactivation [25, 55, 56]. Stress could diminish the levels of CX3CL1/CX3CR1 and CD200/CD200R in hippocampus, and these changes were normalized by antidepressants [57–60]. Consistent with these reports, in this study, we found CMS-induced decrease in the intercommunicating molecules between neuron and microglia (CX3CL1/CX3CR1 and CD200/CD200R) in hippocampus of CMS-exposed mice was reversed by asperosaponin VI treatment. Interestingly, upregulation of CX3CR1 and non-inflammatory microglia were found in post-mortem brain tissue of patients with the major depressive disorder [61], suggesting that the roles of CX3CR1 and microglia-mediated inflammation in depression need to be further investigated.

The alteration of neuron–microglia communication contributes to synaptic function. Our study showed that the PSD-95, CamKII α and CamKII β as well as decreased levels of phosphorylated GluA 2 in hippocampus of CMS-exposed mice. These are thought to be crucial for morphological maturation and synaptic development of hippocampal neurons [62–67]. These changes in synaptic function-related markers were partially reversed by asperosaponin VI, but not in the presence of GW9662. These results suggest that asperosaponin VI requires PPAR-γ to induce neuroprotective microglia and repair CMS-induced damage to synaptic function in hippocampus.

## Conclusion

**Fig. 8:**
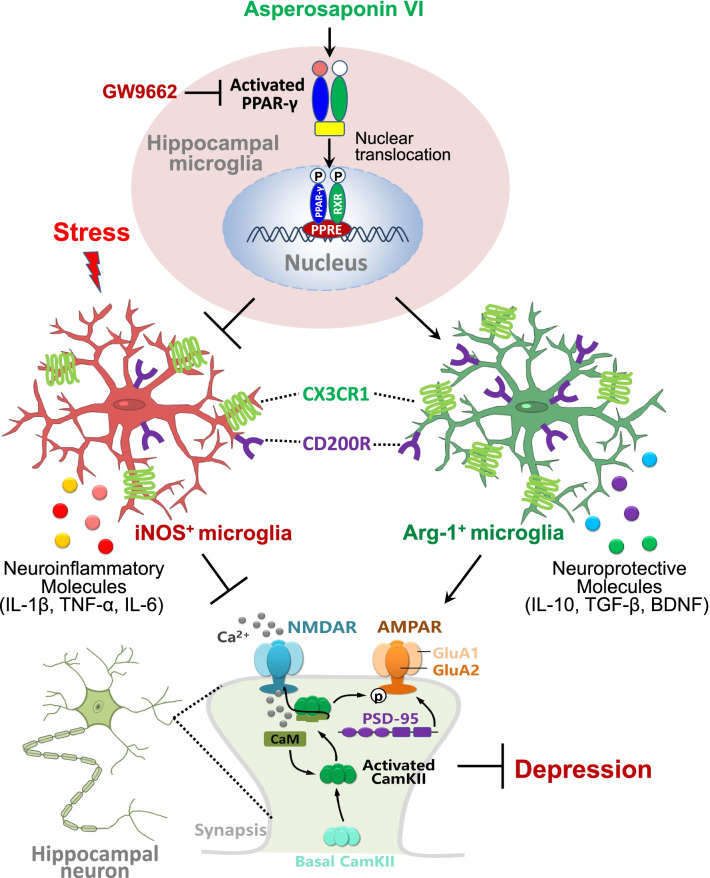
Mechanism by which asperosaponin VI improve CMS-induced depression. Stress induces a pro-inflammatory microglial phenotype (iNOS^+^-Iba1^+^ cells) in hippocampus, that causes reduced crosstalk between microglia and neuron, inflammation and synaptic dysfunction in the hippocampus, ultimately leading to depression-like behaviors. Asperosaponin VI may ameliorate the effects of stress by inducing microglia to adopt a PPAR-γ-dependent anti-inflammatory phenotype (Arg^+^-Iba1^+^ cells) which releasing anti-inflammatory and neuroprotective molecules

In summary, these experiments in mice suggest that CMS induces depression-like behaviors by inducing a pro-inflammatory microglial phenotype that disrupts neuron–microglia communication and synaptic function. Asperosaponin VI ameliorates the effects of CMS by inducing, via PPAR-γ, a neuroprotective microglial phenotype that partially restores hippocampal synaptic function (Fig. 8). These findings may provide further insights into the pathogenesis of depression and the development of natural antidepressants. The present study provides further evidence for asperosaponin VI as a potential antidepressant and a reference for research on depression.

## Supplementary Information

**Additional file 1.** Supplementary materials.

## Data Availability

The raw data supporting the conclusions of this article will be made available by the authors, without undue reservation.

## Abbreviations

AMPAR: α-Amino-3-hydroxy-5-methyl-4-isoxazole-propionic acid receptor
Arg-1: Arginase-1
ASA VI: Asperosaponin VI
BDNF: Brain-derived neurotrophic factor
CamKIIα: α Subunit of calcium/calmodulin-dependent protein kinase II
CamKIIβ: β Subunit of calcium/calmodulin-dependent protein kinase II
CMS: Chronic mild stress
CNS: Central nervous system
DAPI: 4′,6-Diamidino-2-phenylindole
DG: Dentate gyrus
ELISA: Enzyme-linked immunosorbent assay
FSF: Forced swimming test
Iba1: Ionized calcium-binding adapter molecule 1
IL-1β: Interleukin-1 beta
IL-6: Interleukin-6
IL-10: Interleukin-10
IMI: Imipramine hydrochloride
iNOS: Inducible nitric oxide synthase
MDD: Major depressive disorder
NeuN: Neuronal nuclear antigen
OFT: Open field test
PBS: Phosphate-buffered saline
PFA: Paraformaldehyde
PPAR-γ: Peroxisome proliferator-activated receptor-gamma
PSD-95: Postsynaptic density 95
RT-PCR: Real-time polymerase chain reaction
SPT: Sucrose preference test
TGF-β: Transforming growth factor-beta
TNF-α: Tumor necrosis factor alpha
TST: Tail suspension test
