# Supplementary material for: Asperosaponin VI ameliorates the CMS-induced depressive-like behaviors by inducing a neuroprotective microglial phenotype in hippocampus via PPAR-γ pathway
Source: J Neuroinflammation. 2022 May 24;19:115. doi: 10.1186/s12974-022-02478-y (PMC9131532; doi:10.1186/s12974-022-02478-y)
Supplement: Supplementary file 1 — Additional file 1. Supplementary materials. [file 12974_2022_2478_MOESM1_ESM.pdf]

Supplementary materials

Supplementary figures

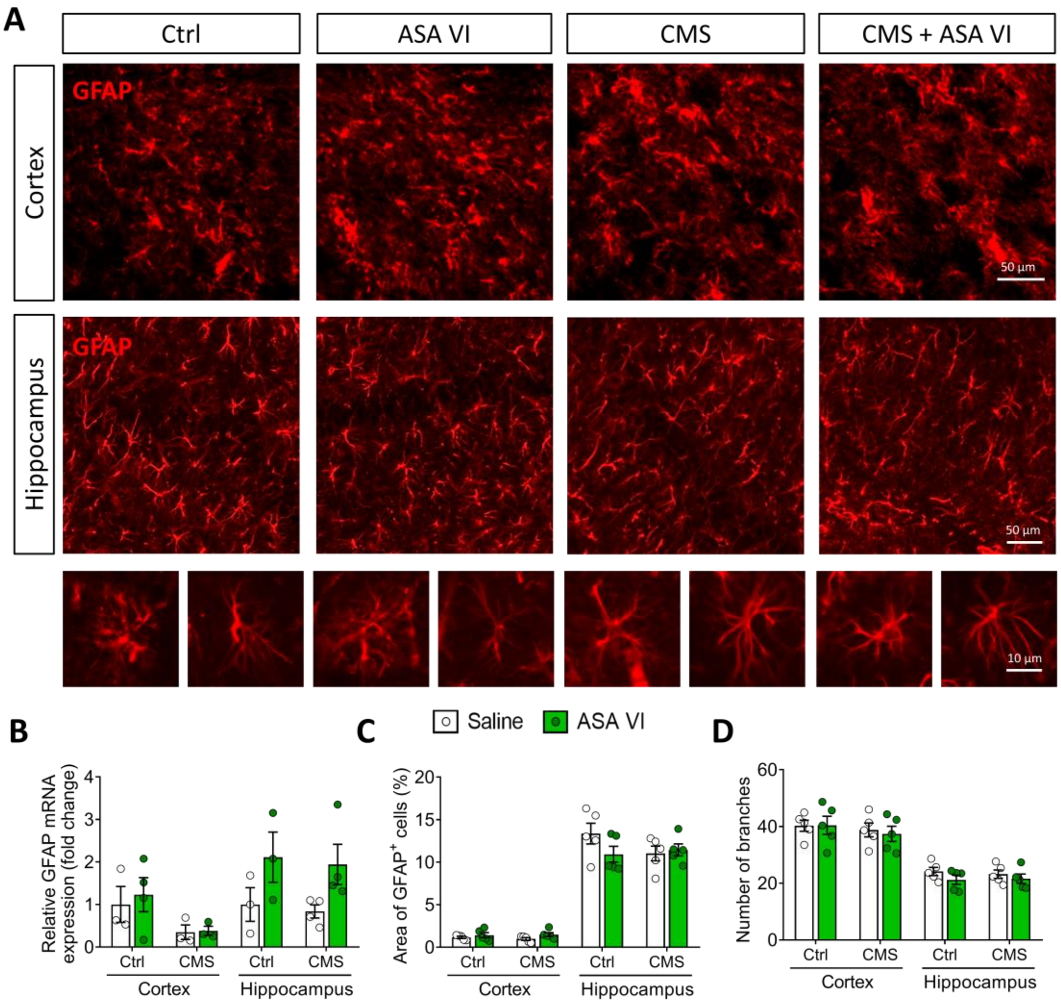

Fig. S1 Effects of asperosaponin VI on the GFAP^{+} cells in cortex and hippocampus of CMS mice

(A) Representative micrographs of astrocytes in hippocampus and cortex of Ctrl or CMS mice after treatment with saline or ASA VI. Astrocytes were stained with GFAP (red) by immunocytochemistry.

(B) Levels of mRNA encoding GFAP in hippocampus and cortex of Ctrl or CMS mice after treatment with saline or ASA VI (n = 4 animals).

(C and D) Quantification of the area and number of branches on GFAP^{+} cells in hippocampus and cortex of Ctrl or CMS mice after treatment with saline or ASA VI (n = 5 animals). Data for individual animals are displayed (mean ± SEM).

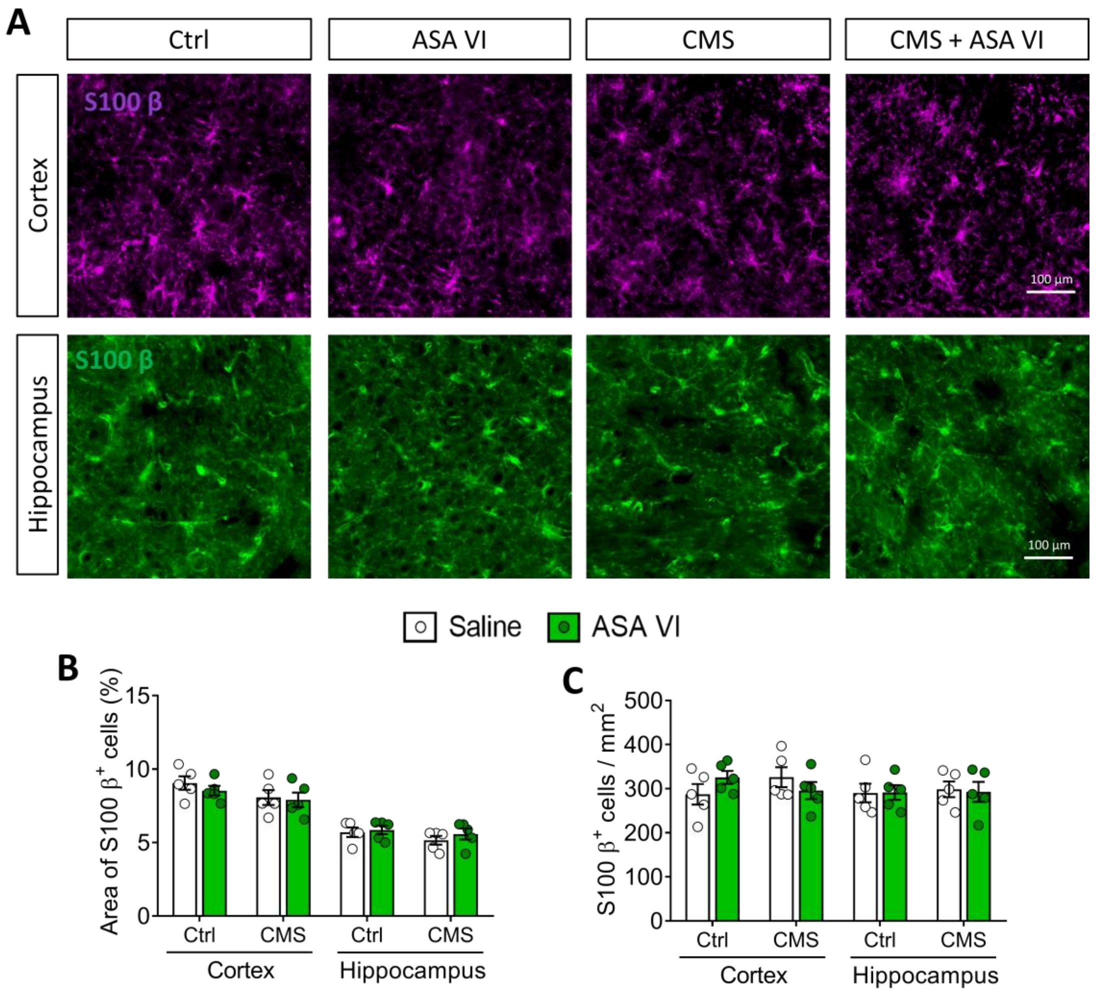

Fig. S2 Effects of asperosaponin VI on the S100β^{+} cells in cortex and hippocampus of CMS mice

(A) Representative micrographs of S100 β^{+} cells in hippocampus and cortex of Ctrl or CMS mice after treatment with saline or ASA VI. Astrocytes were stained with S100 β (purple in cortex and green in hippocampus) by immunocytochemistry.

(B and C) Quantification of the area and number of S100β^{+} cells in hippocampus and cortex of Ctrl or CMS mice after treatment with saline or ASA VI (n = 5 animals). Data for individual animals are displayed (mean ± SEM).

Table S1. The procedure of chronic unpredictable mild stress (CMS)

	Monday	Tuesday	Wednesday	Thursday	Friday	Saturday	Sunday
Week 1	9-21h Continuous dark<br>14-14h Cage tilting<br>21-9h Continuous lighting	9-9:10h Smell stimulation<br>14-14h Damp bedding<br>19-7h Strobe lighting	9-11h Restraint<br>14-14h A soiled cage<br>19-19:15h Tail clipping	19-19h Food deprivation<br>7-19h Water deprivation<br>21-21:10h Smell stimulation	9-21h Continuous dark<br>14-14h Cage tilting<br>19-7h Continuous lighting	9-9:10h Smell stimulation<br>14-14h Damp bedding<br>19-7h Strobe lighting	9-11h Restraint<br>14-14h A soiled cage<br>19-19:15h Tail clipping
Week 2	9-21h Empty water bottles<br>14-15h Cage shaking<br>19-19h Cage tilting	9-21h Continuous dark<br>14-14:10h Smell stimulation<br>21-9h Continuous lighting	9-21h Strobe lighting<br>14-14:15h Tail clipping<br>21-9h Damp bedding	19-19h Food deprivation<br>7-19h Water deprivation<br>21-21h A soiled cage	9-9h Cage tilting<br>14-14:10h Smell stimulation<br>19-21h Restraint	9-21h Continuous dark<br>14-15h Cage shaking<br>21-9h Continuous lighting	9-21h Empty water bottles<br>14-14:15h Tail clipping<br>19-19h Damp bedding
Week 3	9-21h Strobe lighting<br>14-14h Cage tilting<br>19-19h A soiled cage	9-21h Continuous dark<br>14-15h Cage shaking<br>21-9h Continuous lighting	9-21h Empty water bottles<br>14-14:15h Tail clipping<br>19-19:10h Smell stimulation	21-21h Food deprivation<br>7-19h Water deprivation<br>21-23h Restraint	9-9h Cage tilting<br>14-14h Damp bedding<br>19-7h Strobe lighting	9-21h Empty water bottles<br>14-14h A soiled cage<br>19-19:10h Smell stimulation	9-21h Continuous dark<br>14-16h Restraint<br>21-9h Continuous lighting
Week 4	9-9h Damp bedding<br>14-14h Cage tilting<br>19-7h Strobe lighting	9-9h A soiled cage<br>14-14:15h Tail clipping<br>19-7h Empty water bottles	9-21h Continuous dark<br>14-15h Cage shaking<br>21-9h Continuous lighting	21-21h Food deprivation<br>7-19h Water deprivation<br>21-21:10h Smell stimulation	9-11h Restraint<br>14-14h Damp bedding<br>19-19h Cage tilting	9-21h Empty water bottles<br>14-14h A soiled cage<br>19-7h Strobe lighting	9-21h Continuous dark<br>14-14:15h Tail clipping<br>21-9h Continuous lighting
Week 5	9-9h Cage tilting<br>14-14h Damp bedding<br>19-7h Strobe lighting	9-21h Empty water bottles<br>14-14h A soiled cage<br>19-20h Cage shaking	9-21h Continuous dark<br>14-14:10h Smell stimulation<br>21-9h Continuous lighting	21-21h Food deprivation<br>7-19h Water deprivation<br>21-23h Restraint	9-9:15h Tail clipping<br>14-14h Damp bedding<br>19-19h Cage tilting	9-10h Cage shaking<br>14-14h A soiled cage<br>19-19:10h Smell stimulation	9-21h Empty water bottles<br>14-16h Restraint<br>19-7h Strobe lighting
Week 6	9-21h Continuous dark<br>14-14:15h Tail clipping<br>21-9h Continuous lighting	9-9h Cage tilting<br>14-14h Damp bedding<br>19-21h Restraint	9-21h Empty water bottles<br>14-14h A soiled cage<br>19-20h Cage shaking	21-21h Food deprivation<br>7-19h Water deprivation<br>21-21:10h Smell stimulation	9-21h Continuous dark<br>14-16h Restraint<br>21-9h Continuous lighting	9-9h Cage tilting<br>14-14:15h Tail clipping<br>19-7h Strobe lighting	9-9:10h Smell stimulation<br>14-15h Cage shaking<br>19-7h Empty water bottles

Supplementary Table 2. Genes primers used for real time PCR analyses

Gene	Primer sequences
β-actin	Forward: 5’-CCGTGAAAAGATGACCCAGATC-3’<br>Reverse: 5’-CACAGCCTGGATGGCTACGT-3’
Cd11b	Forward: 5’-CACAATGGATGGCTTGATGGA-3’<br>Reverse: 5’-CGTCCACGCAGTCCGGTAAAA-3’
Gfap	Forward: 5’-TGCTGGAGGGCGAAGAAA-3’<br>Reverse: 5’-CGGATCTGGAGGTTGGAGAA-3’
IL-1β	Forward: 5’-CCAGCAGGTTATCATCATCATCC-3’<br>Reverse: 5’-CTCGCAGCAGCACATCAAC-3’
IL-6	Forward: 5-ACCGCTATGAAGTTCCTCTC-3’<br>Reverse: 5’-CTCTGTGAAGTCTCCTCTCC-3’
Tnf-α	Forward:5’-TACTGAACTTCGGGGTGATTGGTCC-3’<br>Reverse: 5’-CAGCCTTGTCCCTTGAAGAGAACC-3’
iNOS	Forward: 5’-ACAACAGGAACCTACCAGCTCA-3’<br>Reverse: 5’-GATGTTGTAGCGCTGTGTGTCA-3’
IL-10	Forward: 5’-TGGCCCAGAAATCAAGGAGC-3’<br>Reverse: 5’-CAGCAGACTCAATACACACT-3’
Tgf-β	Forward: 5’-GACCGCAACAACGCCATCTA-3’<br>Reverse: 5’-GGCGTATCAGTGGGGGTCAG-3’
Arg-1	Forward: 5’-AGACAGCAGAGGAGGTGAAGAG-3’<br>Reverse: 5’-CGAAGCAAGCCAAGGTTAAAGC-3’
Bdnf	Forward: 5’-GAGCTGAGCGTGTGTGACAG-3’<br>Reverse: 5’-CGCCAGCCAATTCTCTTTTTGC-3’
Cx3cr1	Forward: 5’-CAGCATCGACCGGTACCTT- 3’<br>Reverse: 5’-GCTGCACTGTCCGGTTGTT -3’
Cx3cl1	Forward: 5’-ATTGGAAGACCTTGCTTTGG-3’<br>Reverse: 5’-GCCTCGGAAGTTGAGAGAGA-3’
Cd200	Forward: 5’-GGGCATGGCAGCAGTAGCG-3’<br>Reverse: 5’- TGTGCAGCGCCTTTCTTTC-3’
Cd200R	Forward: 5’-AGGAGGATGAAATGCAGCCTTA-3’<br>Reverse: 5’- TGCCTCCACCTTAGTCACAGTATC -3’

Table S3. Details of Antibody and dilution rate for immunofluorescent staining

AntiBody	Manufacturer	Product numbers	Dilution Rate
Iba1 (Goat anti-mouse)	Abcam	ab178846	1:400
GFAP (Rabbit anti-mouse)	Cell Signaling Technology	80788S	1:500
S100β (Rabbit anti-mouse)	Cell Signaling Technology	90393S	1:400
iNOS (Rabbit anti-mouse)	Abcam	ab178945	1:50
Arg-1 (Mouse anti-mouse)	Abcam	ab239731	1:200
PPAR-γ (Rabbit anti-mouse)	Cell Signaling Technology	2435S	1:300
NeuN (Rabbit anti-mouse)	Cell Signaling Technology	36662SF	1:800
CX3CR1 (Rabbit anti-mouse)	Sigma-Aldrich	SAB3500204	1:400
CD200R (Rabbit anti-mouse)	Abcam	ab243839	1:200

Table S4. Details of Antibody and dilution rate for western blotting

Antibody	Manufacturer	Product numbers	Dilution Rate
PSD95 (Goat anti-mouse)	Cell Signaling Technology	36233S	1:800
CaMKII (pan) (Rabbit anti-mouse)	Cell Signaling Technology	4436S	1:800
GAPDH (Mouse anti-mouse)	ab8245	ab8245	1:1000
PPAR-γ (Rabbit anti-mouse)	Cell Signaling Technology	2435S	1:800
p-GluA2 (Rabbit anti-mouse)	ab8245	ab52180	1:600
β-actin (Mouse anti-mouse)	Abcam	ab6276	1:1000
p-PPARγ (S112) (Rabbit anti-mouse)	ImmunoWay Biotechnology	YP0316	1:500
CX3CR1 (Rabbit anti-mouse)	Sigma-Aldrich	SAB3500204	1:800
CD200R (Rabbit anti-mouse)	Abcam	ab243839	1:600
CX3CL1 (Rabbit anti-mouse)	Abcam	ab25088	1:800
CD200 (Rabbit anti-mouse)	Abcam	ab33734	1:800
